# 102 Parous women perform less moderate to vigorous physical activity than their nulliparous peers: a population-based study in Denmark

**DOI:** 10.1093/eurpub/ckae114.177

**Published:** 2024-09-26

**Authors:** Solvej Videbæk Bueno, Rasmus Oestergaard Nielsen, Per Kallestrup, Knud Ryom, Kelly Morgan, Peter Elsborg, Christina Bjørk Petersen, Julie Sandell Jacobsen

**Affiliations:** The Research Unit for General Practice, Aarhus, Denmark; Department of Public Health, Aarhus University, Aarhus, Denmark; The Research Unit for General Practice, Aarhus, Denmark; Department of Public Health, Aarhus University, Aarhus, Denmark; The Research Unit for General Practice, Aarhus, Denmark; Department of Public Health, Aarhus University, Aarhus, Denmark; Department of Public Health, Aarhus University, Aarhus, Denmark; Centre for Development, Evaluation, Complexity and Implementation in Public Health Improvement (DECIPHer), Cardiff University, Cardiff, United Kingdom; Center for Clinical Research and Prevention, Copenhagen University Hospital, Copenhagen, Denmark; National Institute of Public Health, Faculty of Health Science, University of Southern Denmark, Copenhagen, Denmark; The Research Unit for General Practice, Aarhus, Denmark; Research Centre for Health and Welfare Technology, VIA University College, Aarhus, Denmark

## Abstract

**Purpose:**

The World Health Organisation (WHO) highlights parous women as a key population for monitoring trends of physical activity (PA). We aimed to estimate the proportion of Danish women non-adhering to WHO PA guidelines in parous women compared with nulliparous women, and to describe leisure-time PA intensity in each of these groups.

**Methods:**

This population-based study builds on a sample of 27,668 women aged 16-40 years from the Danish National Health Survey 2021. These data were linked with childbirth data from the Danish National Birth Registry. The primary outcome was self-reported weekly hours of moderate to vigorous leisure-time PA (MVPA) dichotomized into: (i) Adhering to WHO guidelines for MVPA, or (ii) Not adhering to WHO guidelines for MVPA. Binomial regression analysis was used to calculate prevalence proportions (PP) and prevalence proportion ratios (PPR).

**Results:**

Of the 27,668 women, a total of 20,022 were included; 9,338 (46.6%) parous women and 10,684 (53.4%) nulliparous women. The PP of women non-adhering to WHO PA guidelines was 63.8% (95% CI 62.9-64.8) for parous and 51.3% (95% CI 50.4-52.3) for nulliparous women, corresponding to a PPR of 1.24 (95% CI 1.21;1.27).

**Conclusions:**

The proportion of parous women who did not adhere to WHO PA guidelines for MVPA was 24% higher than that of nulliparous women. This highlights parous women as a subgroup of the adult population at increased risk of non-adherence to WHO PA guidelines. These findings call for future research to inform new strategies aiming to promote PA in parous women.

**Support/Funding:**

This work was supported by the Health Foundation (20-B-0260).

